# Direct toll-like receptor 4 inhibition in Alzheimer’s disease models: a focused scoping review of inflammatory, amyloid-handling, and functional outcomes

**DOI:** 10.3389/fnagi.2026.1889254

**Published:** 2026-08-17

**Authors:** Xiaolu Ren, Xinyu Jin, Ling Li, Fang Li

**Affiliations:** 1 Department of Radiology, General Hospital of Ningxia Medical University, Yinchuan, Ningxia, China; 2 School of Health Sciences, Universiti Sains Malaysia, Kubang Kerian, Malaysia; 3 Key Laboratory of Respiratory Disease of Zhejiang Province, Department of Respiratory and Critical Care Medicine, The Second Affiliated Hospital, Zhejiang University School of Medicine, Hangzhou, Zhejiang, China; 4 Department of Hematology, General Hospital of Ningxia Medical University, Yinchuan, Ningxia, China; 5 Department of Neurology, General Hospital of Ningxia Medical University, Yinchuan, Ningxia, China

**Keywords:** Alzheimer’s disease, amyloid-β, APOE4, IAXO-101, microglia, neuroinflammation, scoping review, toll-like receptor 4

## Abstract

**Objective:**

This focused scoping review evaluated whether direct toll-like receptor 4 (TLR4) inhibition, antagonism, or genetic suppression in Alzheimer’s disease (AD)-related models was associated with concordant inflammatory, amyloid-β (Aβ)-handling, and functional outcomes.

**Methods:**

Original AD- or Aβ-related animal, cellular, and *ex vivo* studies were eligible when they directly inhibited, antagonized, or genetically suppressed TLR4 signaling using pharmacological approaches, including TAK-242/CLI-095/resatorvid, IAXO-101, RSLA, or related TLR4-targeting strategies, or genetic approaches such as TLR4 siRNA, knockdown, knockout, or loss-of-function mutation. Evidence was synthesized using a three-layer framework comprising inflammatory activation, Aβ handling, and neuronal, synaptic, or behavioral consequences.

**Results:**

Direct TLR4 suppression generally attenuated target-proximal inflammatory activation, including microglial activation, pro-inflammatory mediator expression, NF-κB signaling, NLRP3 inflammasome activation, and reactive microglial phenotypes. In contrast, amyloid-related and functional outcomes were heterogeneous. Some studies reported reduced Aβ pathology, enhanced phagocytosis, synaptic protection, or cognitive improvement, whereas others showed impaired amyloid clearance, increased Aβ deposition, or worsened memory-related outcomes. APOE genotype, sex, treatment window, model system, Aβ species, and intervention type appeared to modify the direction of downstream effects.

**Conclusion:**

Toll-like receptor 4 suppression relatively consistently attenuated inflammatory readouts, whereas amyloid-related and functional outcomes remained heterogeneous, supporting an exploratory, hypothesis-generating model of context-dependent inflammatory-amyloid-functional dissociation rather than a uniform therapeutic benefit.

## 1 Introduction

Alzheimer’s disease (AD) is characterized by progressive cognitive decline accompanied by amyloid-β (Aβ) deposition, tau pathology, synaptic dysfunction, and neuroinflammatory activation. Among innate immune mechanisms, microglia are particularly important because they can either amplify neuroinflammatory injury through cytokine and cytotoxic mediator release or contribute to Aβ recognition, uptake, and clearance (Tahara et al., 2006; Leng and Edison, 2021). This dual function makes microglia-directed immune modulation therapeutically attractive but biologically difficult to interpret. Because TLR4-related microglial activation may participate in both inflammatory amplification and Aβ handling, direct suppression of TLR4 signaling may reduce inflammatory injury without necessarily improving, and in some contexts potentially impairing, amyloid clearance or functional outcomes (Song et al., 2011; Doens and Fernández, 2014).

Toll-like receptor 4 (TLR4) is a key pattern-recognition receptor involved in Aβ-related innate immune activation. Fibrillar Aβ can stimulate microglial activation through receptor complexes involving CD14, TLR2, and TLR4 (Reed-Geaghan et al., 2009). Downstream, TLR4-related signaling has been linked to MyD88/TRIF-dependent inflammatory pathways and to crosstalk with NLRP3 inflammasome and complement signaling, thereby contributing to cytokine release and neuroinflammatory amplification (Yang et al., 2020). Accordingly, TLR4 has been proposed as a potential therapeutic target in AD (Zhou et al., 2020).

Direct TLR4 inhibition, however, raises a critical interpretative problem. Although inhibition of TLR4-related signaling may suppress Aβ-induced inflammatory activation (Liu et al., 2020; Zhu et al., 2024), TLR4-dependent microglial responses may also contribute to Aβ uptake and clearance (Tahara et al., 2006). Thus, reducing TLR4 activity may attenuate neuroinflammation without necessarily improving amyloid pathology or cognitive outcomes. In some experimental contexts, disruption of TLR4 signaling has been associated with increased Aβ deposition and worsened cognitive impairment (Song et al., 2011). This challenges a common assumption in AD neuroinflammation research: that suppression of an upstream inflammatory receptor is sufficient evidence of disease-modifying benefit.

This issue is particularly relevant because direct TLR4 inhibition has been examined in preclinical AD-related settings using pharmacological inhibitors such as TAK-242/CLI-095/resatorvid and genetic approaches such as TLR4 knockdown, knockout, or mutation (Song et al., 2011; Cui et al., 2020; Zhu et al., 2024). Individual studies support the ability of TLR4-directed interventions to modulate inflammatory signaling (Cui et al., 2020; Zhu et al., 2024), but behavioral and amyloid-related consequences appear to depend on model context, timing, and co-interventions. For example, TAK-242 attenuated the memory-facilitating effect of JQ1 in an Aβ-induced AD model (Ahangaran et al., 2026).

Previous reviews have discussed TLR4 signaling in AD from broader perspectives, including TLR4 crosstalk with NLRP3 inflammasome and complement pathways, TLR4 as a therapeutic target, and HMGB1/RAGE/TLR4-mediated inflammatory signaling (Yang et al., 2020; Zhou et al., 2020; Paudel et al., 2020). However, these reviews generally examine TLR4 within broad inflammatory or multi-target therapeutic networks. Less attention has been given to direct TLR4 inhibition as a distinct intervention category. This distinction is important because evidence derived from TLR4 expression changes or multi-target anti-inflammatory interventions cannot determine whether direct TLR4 blockade itself produces disease-relevant benefit.

To address this gap, this focused scoping review synthesizes preclinical evidence on direct pharmacological inhibition, antagonism, or genetic suppression of TLR4 signaling in AD-related models. Rather than treating TLR4 inhibition solely as an anti-inflammatory intervention, we organize the evidence into three outcome layers: inflammatory activation, Aβ pathology or amyloid handling, and cognitive or functional consequences. By separating these layers, this review aims to map whether inflammatory improvement is concordant with downstream amyloid-related and functional outcomes, and to examine whether cross-layer discordance reflects context-dependent inflammatory-amyloid-functional dissociation rather than simple experimental inconsistency.

## 2 Methods

### 2.1 Review design

This study was designed as a focused scoping review with narrative synthesis. The aim was to map preclinical evidence on direct pharmacological inhibition, antagonism, or genetic suppression of TLR4 signaling in AD-related models and to examine whether suppression of TLR4-mediated inflammatory signaling was consistently associated with favorable amyloid-related, neuronal, synaptic, or functional outcomes.

A scoping rather than meta-analytic approach was adopted because the available evidence was heterogeneous in model systems, TLR4-targeting strategies, Aβ preparations, treatment timing, dosing regimens, and outcome measures. The review was conducted in accordance with the principles of the PRISMA extension for Scoping Reviews where applicable (Tricco et al., 2018). A completed PRISMA-ScR checklist, including protocol and registration status, is provided as Supplementary Table 1, and the study-selection process is summarized using a PRISMA 2020-style flow diagram (Page et al., 2021).

### 2.2 Eligibility criteria

Studies were eligible if they met all of the following criteria: (1) they were original experimental studies; (2) they used AD-related animal, cellular, or *ex vivo* models, including Aβ-induced, APP/PS1, EFAD, or other AD-relevant experimental systems; (3) they directly inhibited, antagonized, or genetically suppressed TLR4 signaling; and (4) they reported at least one inflammatory, amyloid-related, neuronal, synaptic, or behavioral outcome.

Eligible TLR4-targeting strategies were classified according to intervention type. These included pharmacological inhibition or antagonism using agents such as TAK-242, CLI-095/resatorvid, IAXO-101, and RSLA; genetic loss-of-function approaches, including TLR4 loss-of-function mutation or knockout; gene-silencing approaches, including TLR4 siRNA or knockdown; and pathway-validation strategies when TLR4 manipulation was used primarily to confirm pathway involvement. Other direct TLR4 antagonistic strategies were also considered eligible when the intervention specifically targeted TLR4 signaling. These intervention categories were charted separately because genetic suppression and pharmacological inhibition may differ in timing, reversibility, compensatory adaptation, and biological interpretation.

Studies were excluded if they were reviews, editorials, conference abstracts, book chapters, purely computational studies, non-original articles, or studies not using AD- or Aβ-related models. Studies were also excluded if they used only non-specific anti-inflammatory agents, natural compounds, or multi-target interventions without direct TLR4 inhibition, antagonism, or genetic suppression. Articles in which TLR4 was measured only as a downstream marker, without direct manipulation of TLR4 signaling, were not included in the core synthesis.

### 2.3 Search strategy and study selection

PubMed, Web of Science Core Collection, Scopus, and Embase were searched from database inception to March 21, 2026. Search terms combined concepts related to Alzheimer’s disease, amyloid-β, TLR4, direct TLR4 inhibition or suppression, neuroinflammation, inflammasome activation, and cognitive or functional outcomes. The complete database-specific search strategies are provided in Supplementary Table 2.

All retrieved records were exported and organized for screening. Duplicate records were identified using DOI, title, database identifier, and manual verification. After duplicate removal, records were screened by title and abstract. Reports were excluded at this stage if they represented non-original publication types or were clearly outside the focused AD/Aβ-direct TLR4 review scope.

**FIGURE 1:**
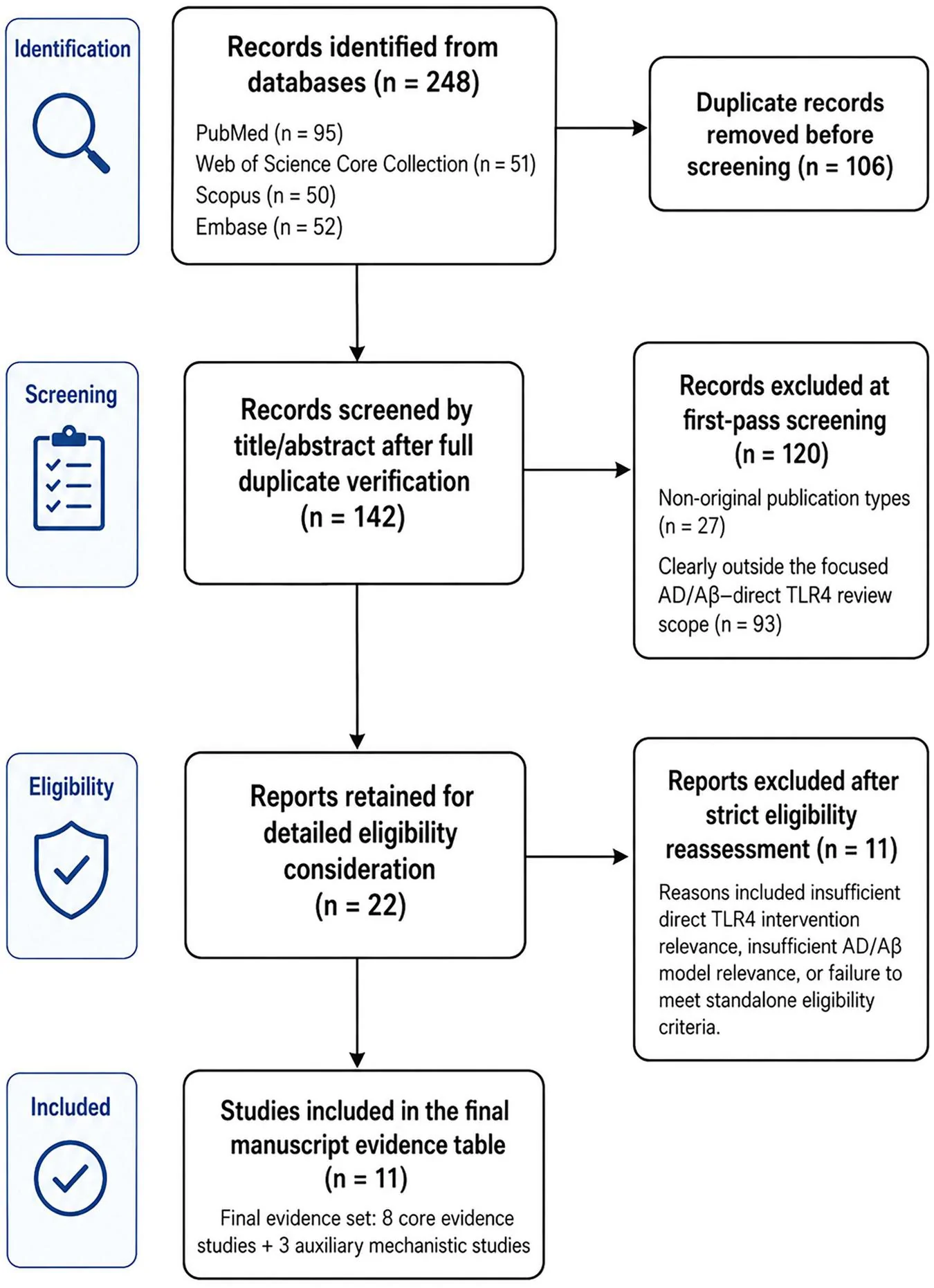
PRISMA flow diagram of study selection.

Potentially relevant reports were retained for detailed eligibility consideration. Eligibility was reassessed according to whether each report used an AD- or Aβ-related model and whether direct TLR4 inhibition, antagonism, genetic suppression, or pathway validation was central to the experimental design. Screening and data charting were conducted using predefined criteria and extraction items, and eligibility decisions were verified by the review team. The PRISMA numerical sequence after strict eligibility reassessment is provided in Supplementary Table 3a, and detailed deduplication, screening, exclusion, and final included-study records are provided in Supplementary Tables 3b–e. The study-selection process is summarized in Figure 1.

### 2.4 Data charting and evidence classification

For each included study, data were charted using a standardized extraction framework. Extracted information included first author, publication year, model type, experimental system, TLR4-targeting strategy, intervention timing, sex, APOE genotype when applicable, inflammatory outcomes, amyloid-related outcomes, neuronal or synaptic outcomes, behavioral or functional outcomes, and the main conclusions relevant to TLR4 signaling.

Studies were interpreted according to evidence directness. Studies were considered higher-directness evidence when direct pharmacological inhibition, antagonism, or genetic suppression of TLR4 was a central experimental intervention and when the findings informed inflammatory, amyloid-related, neuronal/synaptic, or functional outcome domains. *In vivo* studies with cross-layer outcome assessment were considered particularly informative for evaluating whether inflammatory effects were concordant with downstream amyloid-related or functional outcomes.

Cellular, *ex vivo*, and pathway-validation studies were retained as supportive mechanistic evidence. This included studies in which TLR4 manipulation was used mainly to verify pathway involvement, studies in which the primary intervention was not a TLR4-targeting strategy, or studies limited to cellular or *ex vivo* readouts. These studies were used to clarify TLR4-related mechanisms but were not interpreted as standalone therapeutic-efficacy evidence. Studies in which TLR4 was only measured as a downstream inflammatory marker, without direct pharmacological or genetic manipulation of TLR4 signaling, were not included in the main evidence map and were considered only for contextual interpretation when necessary.

Accordingly, these mechanistic studies were not interpreted as equivalent to *in vivo* therapeutic-efficacy evidence.

### 2.5 Three-layer outcome mapping and narrative synthesis

The included studies were synthesized using a three-layer outcome-mapping framework. This framework was developed to capture the possibility that TLR4 inhibition may produce different effects across inflammatory, amyloid-related, and functional domains.

The first layer was the inflammatory layer, which included microglial or astroglial activation, cytokine production, NF-κB signaling, MyD88-dependent signaling, Rac1-related inflammatory signaling, NLRP3 inflammasome activation, oxidative stress, and inflammatory polarization. The second layer was the amyloid-handling layer, which included Aβ deposition, Aβ accumulation, plaque burden, Aβ uptake, phagocytosis, clearance, or APP-processing-related markers. The third layer was the functional layer, which included cognitive behavior, learning and memory performance, synaptic plasticity, long-term potentiation, neuronal viability, apoptosis, and cell death.

Meta-analysis was not performed because of substantial heterogeneity in experimental models, Aβ species, intervention types, treatment windows, dosing regimens, and outcome measurements. Instead, evidence was synthesized narratively by comparing model characteristics, mode of TLR4 targeting, genetic background, sex, APOE genotype when applicable, timing of intervention, Aβ-related context, and consistency or discordance of outcomes across the three predefined layers.

### 2.6 Protocol, evidence directness, and methodological-context appraisal

No separate review protocol was prospectively registered or deposited in a publicly accessible repository, which is acknowledged as a methodological limitation. However, before formal screening, the review team predefined the main protocol elements, including the review question, eligibility criteria, databases to be searched, search concepts, outcome-mapping framework, data-charting items, and evidence-classification principles. This study was designed as a focused scoping review with systematic searching and narrative evidence mapping rather than as a quantitative efficacy-oriented systematic review or meta-analysis. To enhance transparency and reproducibility, the PRISMA-ScR checklist, complete database-specific search strategies, deduplication process, screening decisions, eligibility-stage exclusion reasons, and final included-study records are provided in Supplementary Tables 1–3.

Because the review aimed to map evidence rather than estimate pooled effects, the methodological appraisal was used to contextualize interpretation rather than to exclude studies or statistically weight findings. For *in vivo* animal studies, we added a SYRCLE/CAMARADES-informed methodological appraisal covering randomization, allocation concealment, blinding, sample-size justification, attrition reporting, selective outcome reporting, clarity of intervention timing and dose, animal sex, genotype reporting, and conflict-of-interest reporting. For *in vitro*, *ex vivo*, and cell-based studies, a separate custom critical appraisal framework was used because animal risk-of-bias tools are not directly applicable to these designs. This framework considered model description, Aβ preparation or context, intervention specificity, dose and exposure timing, control conditions, replication, and limits of therapeutic inference.

The appraisal results are provided in Supplementary Table 4 and were used to interpret evidence confidence and methodological context. Accordingly, *in vivo* studies with direct TLR4-targeted intervention and cross-layer outcome assessment were interpreted as higher-directness evidence, whereas cellular, *ex vivo*, and pathway-validation studies were treated as supportive mechanistic evidence rather than standalone therapeutic-efficacy evidence.

## 3 Results

### 3.1 Study selection

A total of 248 records were identified from four databases: PubMed (*n* = 95), Web of Science Core Collection (*n* = 51), Scopus (*n* = 50), and Embase (*n* = 52). After removal of duplicate records by DOI, title, database identifier, and manual verification, 142 records remained for title and abstract screening.

During first-pass title and abstract screening, 27 records were excluded because they were non-original publication types, including reviews, meta-analyses, case reports, conference abstracts or papers, book chapters, preprints, abstracts, and other non-primary evidence. A further 93 records were excluded because they were clearly outside the focused AD/Aβ-direct TLR4 review scope. Therefore, 22 reports were retained for detailed eligibility consideration.

After strict eligibility reassessment, 11 reports were excluded because direct TLR4 intervention, AD/Aβ-core model relevance, or standalone eligibility was insufficient. Finally, 11 studies were included in the manuscript evidence tables. The final evidence set comprised eight core evidence studies and three auxiliary mechanistic studies. The PRISMA numerical sequence is provided in Supplementary Table 3a, and the study-selection process is shown in Figure 1.

### 3.2 Characteristics of included studies

The 11 included studies were published between 2011 and 2026 and covered a broad range of AD- or Aβ-related experimental systems, including APP/PS1 or EFAD transgenic mouse models, acute Aβ-induced rodent models, BV2 and primary microglia, primary hippocampal neurons, astrocyte-related systems, hippocampal slices, and neuron-astrocyte co-cultures. Acute Aβ injection or stimulation models were interpreted as short-term amyloid-induced toxicity, inflammatory activation, or synaptic-response models, whereas APP/PS1 and EFAD models were interpreted as chronic transgenic models of progressive amyloid accumulation and multicellular neuroimmune interactions.

Toll-like receptor 4-targeting or pathway strategies included genetic TLR4 loss-of-function mutation, TLR4 knockout, TLR4 knockdown, TLR4 siRNA, TLR4 overexpression-based validation, and pharmacological inhibition or antagonism using TAK-242, CLI-095/resatorvid, Cyp, RSLA, and IAXO-101. One Aβ1-42-induced rodent study also examined TAK-242 alone and in combination with the BRD4 inhibitor JQ1.

Toll-like receptor 4-targeting or pathway strategies were stratified by intervention type. Pharmacological inhibition or antagonism included TAK-242, CLI-095/resatorvid, Cyp, RSLA, and IAXO-101. Genetic approaches included TLR4 loss-of-function mutation, TLR4 knockout, TLR4 knockdown, and TLR4 siRNA. Pathway-validation approaches included TLR4 overexpression-based validation or downstream pathway interrogation. One Aβ1-42-induced rodent study also examined TAK-242 alone and in combination with the BRD4 inhibitor JQ1. These categories were interpreted separately because pharmacological blockade and genetic suppression do not necessarily represent equivalent biological interventions.

According to the evidence-directness framework summarized in Table 1, *in vivo* studies with direct TLR4-targeted intervention and cross-layer outcome assessment were interpreted as higher-directness evidence. Cellular, *ex vivo*, and pathway-validation studies were retained as supportive mechanistic evidence and were not interpreted as standalone therapeutic-efficacy evidence. Overall, Table 1 defines the evidence role of each study and distinguishes core *in vivo* evidence from auxiliary mechanistic or pathway-validation evidence.

**TABLE 1:** Characteristics and evidence directness of the 11 included studies on direct TLR4 inhibition, antagonism, genetic suppression, or pathway validation in AD-related models.

No.	Study	AD-related model	Intervention category	Direct TLR4-targeting or pathway strategy	Experimental design and outcomes assessed	Role in review/evidence directness
1	Song et al., 2011	APPswe/PS1dE9 mice with TLR4 wild-type or TLR4 loss-of-function mutation	Genetic loss-of-function	Genetic TLR4 loss-of-function	Compared TLR4-mutant and TLR4-wild-type APP/PS1 mice, mainly at 5 and 9 months. Outcomes included microglial activation, Aβ deposition, fibrillar Aβ, soluble Aβ42, cytokine/chemokine readouts, and Morris water maze performance.	Core *in vivo* paradox evidence. Reduced inflammatory or microglial readouts coincided with increased later amyloid burden and impaired spatial acquisition.
2	Wu et al., 2015	Primary rat hippocampal neurons with LPS-induced TLR4/NF-κB activation and APP-processing/Aβ1–42 readouts	Pharmacological pathway probing	CLI-095; NF-κB inhibitor PDTC	Used CLI-095 and PDTC to probe the neuronal TLR4/NF-κB contribution to APP-processing and Aβ1–42 secretion. Outcomes included TNF-α, IL-1β, Aβ1–42 secretion, ADAM10, BACE-1, PS-1, β-APP, and TLR4 expression.	Supportive neuronal mechanistic evidence only; no *in vivo* AD-model validation, amyloid-plaque or clearance assessment, or behavioral evidence.
3	Balducci et al., 2017	Acute Aβ oligomer-induced mouse model; C57BL/6 and TLR4 knockout mice	Pharmacological antagonism + genetic validation	Cyp TLR4 antagonist; TLR4 knockout	Assessed TLR4 dependence of AβO-induced glial activation and memory-establishment impairment. Outcomes included glial activation, IL-1β-associated neuroinflammation, and novel object recognition memory.	Core acute *in vivo* functional evidence. Supports TLR4-dependent glial activation in acute soluble AβO-induced memory-establishment impairment; chronic amyloid handling was not assessed.
4	Cui et al., 2020	APP/PS1 mice; Aβ25–35-induced BV2 microglia	Pharmacological inhibition + pathway validation	TAK-242; NLRP3-siRNA for pathway validation	Tested TAK-242 *in vivo* and *in vitro*; used NLRP3-siRNA to examine pathway dependence. Outcomes included M1/M2 markers, iNOS, TNF-α, TREM-2, Arg-1, Bax, MyD88/NF-κB/NLRP3 signaling, BV2 phagocytosis, and Morris water maze performance.	Core *in vivo* pharmacological evidence. TAK-242 improved inflammatory, amyloid, neuronal-injury, and behavioral layers in an APP/PS1 context.
5	Liu et al., 2020	BV2 and primary microglia; HT-22 cells exposed to microglia-conditioned medium	Pharmacological inhibition; cellular pathway study	CLI-095	LPS-primed microglia were pretreated with CLI-095 and then stimulated with Aβ1–42; conditioned medium was used to assess neuronal toxicity in HT-22 cells. Outcomes included NLRP3, ASC, caspase-1, IL-1β, Iba-1, inflammatory-factor mRNA, HT-22 viability, and apoptosis.	Supportive microglial mechanistic evidence only; no *in vivo* amyloid or behavioral endpoints.
6	Hughes et al., 2020	BV2 cells, astrocytes, TLR4/MyD88 knockout systems, hippocampal slices, and neuron–astrocyte co-cultures	Pharmacological antagonism/ inhibition + genetic validation	RSLA; TAK-242; TLR4/MyD88 knockout	Used soluble Aβ42 aggregates to test TLR4/MyD88-dependent inflammatory, synaptic, and cell-death responses. Outcomes included TNF-α, IL-1β-related signaling, LTP impairment, and neuron/astrocyte cell death.	Supportive mechanistic-functional evidence. Demonstrates compound- and compartment-dependent dissociation but not *in vivo* therapeutic efficacy.
7	Chen et al., 2021	Aβ-stimulated BV2 microglial cells	Pharmacological pathway validation	TAK-242 pathway validation; oxysophoridine primary intervention	Oxysophoridine was the primary intervention; TAK-242 was used to verify TLR4/NF-κB pathway involvement. Outcomes included BV2 viability, oxidative stress, inflammatory mediators, and TLR4/MyD88/NF-κB activation.	Supportive pathway-validation evidence only; not standalone evidence of direct TLR4-inhibitor efficacy.
8	Balu et al., 2023	EFAD mice carrying familial AD mutations and human APOE3/APOE4; sex- and genotype-stratified	Pharmacological antagonism	IAXO-101 CD14/TLR4 antagonist	Prevention and reversal paradigms; subcutaneous vehicle-controlled treatment across APOE, sex, and treatment-window contexts. Outcomes included Iba-1, reactive microglia, GFAP, Clec7a, IL-1β, Aβ deposition, fibrillar amyloid, soluble/insoluble Aβ and apoE, and modified Morris water maze performance.	Core context-dependent *in vivo* evidence. Effects varied by sex, APOE genotype, and treatment window.
9	Dou et al., 2024	Aβ1–42-induced BV2 microglial AD-like cell model	Genetic knockdown/ overexpression	Lentiviral TLR4 knockdown and overexpression	Manipulated TLR4 to test its causal role in Aβ-induced BV2 cellular injury. Outcomes included inflammatory cytokines, NLRP3/caspase-1, Bax/Bcl-2, apoptosis, and BV2 tau-related proteins.	Supportive genetic cell-model evidence only; BV2 tau-related changes were not interpreted as neuronal tau pathology or cognitive evidence.
10	Zhu et al., 2024	Aβ1–42-treated BV2 cells; APP/PS1 mice	Gene silencing + pharmacological/ pathway inhibition	TLR4 siRNA; CLI-095; Rac1 inhibitor NSC23766	Examined the Aβ–TLR4–Rac1–NLRP3 axis in cells and APP/PS1 mice. Outcomes included Rac1 activation, NLRP3 inflammasome, microglial activation, cytokines, Aβ deposition, and Morris water maze performance.	Core *in vivo* pathway evidence. Direct TLR4 inhibition improved inflammatory and behavioral outcomes, whereas Aβ-deposition reduction was demonstrated mainly after downstream Rac1 inhibition; amyloid claims should therefore be attributed conservatively.
11	Ahangaran et al., 2026	Male Wistar rat model induced by bilateral intracerebroventricular Aβ1–42 injection	Pharmacological inhibition + co-intervention	TAK-242 alone or with BRD4 inhibitor JQ1	Compared saline, Aβ, Aβ+JQ1, Aβ+TAK-242, and Aβ+JQ1+TAK-242 groups. Outcomes included passive avoidance, Morris water maze, CD86/CD163 microglial index, and hippocampal BDNF/proBDNF.	Core discordant *in vivo* evidence. Selected microglial improvement after TAK-242 did not consistently translate into BDNF-related or spatial-memory benefit, and TLR4 inhibition attenuated JQ1-related memory facilitation.

Evidence directness was classified according to model type, TLR4-targeting role, and outcome coverage. Cellular, *ex vivo*, and pathway-validation studies were considered supportive mechanistic evidence rather than standalone therapeutic-efficacy evidence. Intervention categories were assigned according to the primary mode of TLR4 targeting or pathway interrogation and were not treated as biologically interchangeable.

### 3.3 Outcome-direction matrix and evidence classification

All 11 included studies were incorporated into the outcome-direction matrix and evidence-level synthesis. Table 2 summarizes the direction of inflammatory or microglial, amyloid-related, neuronal/synaptic/trophic, and functional or behavioral outcomes after TLR4 inhibition, antagonism, genetic suppression, or pathway validation. Table 3 further maps each study onto a three-layer framework comprising target-proximal inflammatory or microglial signaling, intermediate amyloid-related or neurocellular outcomes, and distal functional or behavioral outcomes.

**TABLE 2:** Outcome-direction matrix after TLR4 inhibition, antagonism, genetic suppression, or pathway validation in AD-related models.

No.	Study/approach/evidence role	Inflammatory or microglial outcome	Amyloid-related outcome	Neuronal/synaptic/trophic outcome	Functional/behavioral outcome	Conservative overall interpretation
1	Song et al., 2011; TLR4 loss-of-function; core *in vivo* paradox	Decreased selected microglial/cytokine readouts.	Increased later Aβ deposition, fibrillar Aβ, and soluble Aβ42.	Not reported.	Worsened Morris water maze acquisition; probe trial not clearly changed.	Inflammation improved while amyloid burden and acquisition learning worsened.
2	Wu et al., 2015; CLI-095/PDTC; supportive neuronal mechanism	Decreased LPS-induced neuronal TNF-α and IL-1β; microglia not assessed.	CLI-095/PDTC reversed LPS-induced ADAM10 downregulation, BACE-1/PS-1 upregulation, β-APP increase, and Aβ1–42 secretion in neurons; no plaque-burden or Aβ-clearance data.	Not reported.	Not reported.	Supports a neuronal TLR4/NF-κB–APP-processing link only; no *in vivo* amyloid-clearance or therapeutic-efficacy inference.
3	Balducci et al., 2017; Cyp/KO; core acute *in vivo* function	Decreased acute AβO-induced glial activation and IL-1β-associated inflammation.	Not reported for plaque burden or Aβ clearance.	Not reported.	Improved AβO-induced novel object recognition memory-establishment impairment.	Benefit applies to acute AβO-driven inflammatory memory impairment, not chronic amyloid handling.
4	Cui et al., 2020; TAK-242; core pharmacological *in vivo*	Decreased microglial activation, M1 markers, and MyD88/NF-κB/NLRP3 signaling; increased M2/phagocytic markers.	Decreased Aβ plaque load; BV2 bead phagocytosis increased, but direct Aβ phagocytosis was not specifically quantified.	Reduced Bax/DNA fragmentation and conditioned-medium-induced neuronal apoptosis.	Improved Morris water maze performance.	Beneficial cross-layer pattern in APP/PS1 mice.
5	Liu et al., 2020; CLI-095; supportive microglial mechanism	Decreased Aβ1–42-induced NLRP3/ASC/caspase-1/IL-1β and inflammatory mediators.	Not reported.	Reduced microglia-conditioned-medium neurotoxicity toward HT-22 cells.	Not reported.	Supports cellular TLR4/NLRP3-mediated microglial neurotoxicity pathway.
6	Hughes et al., 2020; RSLA/TAK-242/KO; supportive *ex vivo* mechanism-function	Decreased TLR4/MyD88-dependent cytokine responses; antagonists blocked glial TNF-α in culture.	Not reported.	Mixed: RSLA rescued Aβ-induced LTP impairment, whereas TAK-242 did not; antagonists reduced co-culture cell death.	No animal behavior; LTP was used as an *ex vivo* functional readout.	Shows synaptic/cell-death effects are compound- and compartment-dependent.
7	Chen et al., 2021; TAK-242 validation; oxysophoridine primary	TAK-242-sensitive reduction in TLR4/MyD88/NF-κB, cytokines, and oxidative stress in BV2 cells.	Not reported.	BV2 viability/oxidative stress assessed; neuronal pathology not reported.	Not reported.	Pathway-validation evidence only; not direct TLR4-inhibitor efficacy.
8	Balu et al., 2023; IAXO-101; core context-dependent *in vivo*	Decreased most clearly in female E4FAD; limited or absent in male E4FAD and female E3FAD contexts.	Mixed: prevention showed memory benefit without Aβ reduction and increased cortical Thio-S; reversal reduced histological Aβ/fibrillar amyloid.	Not reported.	Improved Morris water maze probe memory in female E4FAD only.	Benefit depends on sex, APOE genotype, and treatment window.
9	Dou et al., 2024; TLR4 knockdown/overexpression; supportive genetic cell mode	TLR4 knockdown decreased BV2 cytokines, NLRP3/caspase-1, Bax, and apoptosis; overexpression showed the opposite pattern.	Not reported for Aβ clearance, plaque burden, or APP processing.	Reduced BV2 tau-related proteins after knockdown; not neuronal tau pathology.	Not reported.	Supports TLR4-dependent BV2 inflammatory, apoptotic, and tau-related cellular changes only.
10	Zhu et al., 2024; TLR4 siRNA/CLI-095/NSC23766; core pathway evidence	Decreased TLR4/Rac1/NLRP3 activation and inflammatory mediator expression in cells and mice.	Reduced hippocampal Aβ deposition was reported after Rac1 inhibition; CLI-095-related Aβ lowering should not be overgeneralized.	Not reported for neuronal loss, synaptic proteins, BDNF, or tau.	Improved Morris water maze performance after CLI-095 or NSC23766.	Supports a targetable Aβ–TLR4–Rac1–NLRP3 inflammatory axis; behavioral improvement was observed after CLI-095 or NSC23766, whereas amyloid-deposition reduction should be attributed mainly to Rac1 inhibition.
11	Ahangaran et al., 2026; TAK-242 ± JQ1; core discordant *in vivo*	Decreased CD86/CD163-based microglial polarization index; canonical TLR4 downstream signaling was not reported.	Not reported.	JQ1 improved mature BDNF-related measures; TAK-242 alone did not consistently rescue BDNF readouts.	Mixed/adverse: JQ1 improved memory outcomes, whereas TAK-242 alone failed to rescue Morris water maze performance and co-treatment attenuated JQ1-related spatial and aversive memory benefits.	Selected microglial improvement after TAK-242 did not guarantee neurotrophic or spatial cognitive rescue; TLR4 inhibition attenuated JQ1-related memory facilitation.

Outcome directions should be interpreted together with evidence directness and intervention category in Table 1. “Mixed” indicates inconsistent directions across models, interventions, or endpoints; “not reported” indicates no direct assessment. Pharmacological inhibition or antagonism, genetic loss-of-function or knockout, gene-silencing approaches, pathway-validation strategies, and combination/co-intervention designs were not treated as biologically interchangeable. For Zhu et al. (2024) amyloid-deposition findings should be attributed to the specific Rac1-inhibition experiment rather than generalized to all TLR4-targeting approaches. For Ahangaran et al. (2026) TAK-242 should not be described as consistently behaviorally beneficial.

**TABLE 3:** Evidence-level synthesis of inflammatory, amyloid-related, and functional outcome patterns after TLR4 inhibition, antagonism, genetic suppression, or pathway validation in AD-related models.

No.	Study	Layer 1: TLR4/inflammatory or microglial signaling	Layer 2: amyloid-related, neuronal, synaptic, or trophic evidence	Layer 3: functional/behavioral evidence	Contribution to inflammatory–amyloid–functional dissociation	Interpretation for Figure 2/hypothesis-generating synthesis
1	Song et al., 2011	TLR4 mutation reduced microglial activation and selected cytokine/chemokine responses.	Increased later Aβ deposition, fibrillar Aβ, and soluble Aβ42.	Impaired Morris water maze acquisition; probe trial not clearly worsened.	Strong paradox evidence: target-proximal inflammatory attenuation coincided with downstream amyloid worsening.	Protective/clearance-side branch: TLR4-dependent microglial activation may support Aβ handling in some stages.
2	Wu et al., 2015	CLI-095/PDTC reduced neuronal inflammatory signaling; microglia not assessed.	CLI-095/PDTC reversed LPS-induced ADAM10 downregulation, BACE-1/PS-1 upregulation, β-APP increase, and Aβ1–42 secretion in primary hippocampal neurons; no plaque or clearance evidence.	Not assessed.	Supportive neuronal mechanism only.	Mechanistic branch: neuronal TLR4/NF-κB activation may promote amyloidogenic APP processing and Aβ1–42 secretion, but this does not establish *in vivo* amyloid-clearance or therapeutic efficacy.
3	Balducci et al., 2017	TLR4 blockade/knockout reduced AβO-induced glial activation and IL-1β-associated inflammation.	Chronic amyloid handling, neuronal loss, synaptic proteins, and BDNF were not assessed.	Prevented AβO-induced novel object recognition memory-establishment impairment.	Beneficial-inhibition evidence in acute soluble AβO context.	Beneficial branch: TLR4 suppression may help when soluble AβO acutely drives inflammatory memory impairment.
4	Cui et al., 2020	TAK-242 reduced M1 markers and MyD88/NF-κB/NLRP3 signaling and increased M2/phagocytic markers.	Reduced Aβ plaques and neuronal apoptosis-related readouts; direct Aβ phagocytosis was not specifically quantified.	Improved Morris water maze performance.	Higher-directness beneficial cross-layer evidence.	Beneficial branch: inhibition helps when maladaptive TLR4/MyD88/NF-κB/NLRP3 signaling dominates.
5	Liu et al., 2020	CLI-095 attenuated Aβ-induced NLRP3/ASC/caspase-1/IL-1β signaling.	Reduced microglia-conditioned-medium neurotoxicity; no Aβ plaque, APP-processing, synaptic, or BDNF data.	Not assessed.	Supportive microglial mechanism only.	Mechanistic arrow: Aβ→ TLR4 → NLRP3/caspase-1/IL-1β→ microglia-mediated neuronal injury.
6	Hughes et al., 2020	Aβ42 oligomers induced TLR4/MyD88 cytokine responses; antagonists blocked glial TNF-α in culture.	RSLA rescued LTP impairment but TAK-242 did not; antagonists reduced co-culture cell death.	No animal behavior; LTP served as an *ex vivo* functional readout.	Mechanistic-functional evidence showing compound/compartment dissociation.	Links soluble Aβ aggregates, TLR4 inflammation, synaptic plasticity failure, and neuron–glia toxicity.
7	Chen et al., 2021	TAK-242 reduced Aβ-induced TLR4/MyD88/NF-κB and inflammatory–oxidative readouts in BV2 cells.	BV2 viability/oxidative stress assessed; Aβ plaque, clearance, APP processing, neuronal pathology, and BDNF were not assessed.	Not assessed.	Pathway-validation evidence only; oxysophoridine was the primary compound.	Cellular pathway branch: Aβ activates microglial TLR4/MyD88/NF-κB signaling.
8	Balu et al., 2023	IAXO-101 reduced microglial reactivity most clearly in female E4FAD mice.	Mixed amyloid effects by prevention/reversal paradigm.	Improved Morris water maze probe memory in female E4FAD only.	Strong context-dependent evidence; effects varied by sex, APOE genotype, and timing.	Precision-context layer: TLR4 antagonism may benefit selected APOE4 inflammatory contexts, not universally.
9	Dou et al., 2024	TLR4 knockdown reduced BV2 cytokines, NLRP3/caspase-1, Bax, and apoptosis; overexpression showed the opposite direction.	Reduced BV2 tau-related protein changes; no Aβ clearance, plaque, APP processing, or *in vivo* pathology.	Not assessed.	Supportive genetic cell-model evidence only.	Cellular branch: Aβ1–42 activates TLR4-dependent microglial inflammatory and apoptotic signaling.
10	Zhu et al., 2024	TLR4 knockdown/CLI-095 reduced TLR4/Rac1/NLRP3 activation and inflammatory mediators.	NSC23766 reduced hippocampal Aβ deposition; CLI-095-related Aβ lowering should not be overgeneralized.	CLI-095 and NSC23766 improved Morris water maze performance.	Higher-directness *in vivo* pathway evidence, but not definitive evidence that direct TLR4 inhibition itself reduces amyloid deposition.	Mechanistic axis: Aβ→ TLR4 → Rac1 → NLRP3 → inflammatory cytokines → cognitive impairment; amyloid-deposition improvement should be attributed conservatively to Rac1 inhibition.
11	Ahangaran et al., 2026	TAK-242 reduced CD86/CD163 index; canonical TLR4 downstream signaling was not quantified.	JQ1 improved BDNF measures; TAK-242 alone did not consistently improve BDNF; Aβ plaque/clearance was not assessed.	JQ1 improved spatial-memory and BDNF-related outcomes, whereas TAK-242 alone did not rescue spatial memory and co-treatment attenuated JQ1-related spatial and aversive memory benefits.	Discordant evidence: selected microglial improvement after TAK-242 did not proportionally translate downstream.	Paradox/dissociation branch: receptor-level TLR4 inhibition may not guarantee neurotrophic or spatial-memory benefit and may interfere with JQ1-related memory facilitation.

The three layers represent inflammatory/microglial signaling, amyloid-related or neurocellular outcomes, and functional/behavioral outcomes. Cross-layer interpretation was based mainly on higher-directness *in vivo* evidence. Cellular, *ex vivo*, and pathway-validation studies were used only to support mechanistic plausibility. The revised interpretation prevents over-attribution of Rac1-inhibition effects to direct TLR4 inhibition and avoids overstating TAK-242 as behaviorally beneficial in Ahangaran et al. (2026).

Outcome directions were interpreted together with the evidence-directness information in Table 1 and the methodological appraisal summarized in Supplementary Table 4, which was used to contextualize evidence confidence rather than to exclude studies or statistically weight findings. Higher-directness *in vivo* studies provided the main basis for cross-layer therapeutic interpretation. In contrast, cellular, *ex vivo*, and pathway-validation studies were used to clarify TLR4-related mechanisms but were not considered equivalent to *in vivo* therapeutic-efficacy, amyloid-pathology, or cognitive evidence. For example, Wu et al. (2015) supported a neuronal TLR4/NF-κB-APP-processing link, Dou et al. (2024) supported TLR4-dependent BV2 inflammatory and apoptotic changes, and Chen et al. (2021) was interpreted as a TAK-242-sensitive TLR4/MyD88/NF-κB pathway-validation study rather than a direct *in vivo* TLR4-inhibitor efficacy study.

Therefore, outcome directions in Tables 2, 3 should be interpreted according to evidence directness rather than as evidence of equivalent therapeutic significance.

### 3.4 Context-dependent inflammatory–amyloid–functional dissociation pattern

When interpreted according to evidence directness, the included studies suggested a context-dependent three-layer dissociation across inflammatory, amyloid-related, and functional outcomes, although the strength of support differed across *in vivo*, *ex vivo*, cellular, and pathway-validation evidence. The most consistent effect of TLR4 suppression was observed at the inflammatory or microglial signaling layer. When directly measured, TLR4 inhibition, antagonism, or genetic suppression generally reduced target-proximal inflammatory readouts, including TLR4/MyD88/NF-κB signaling, NLRP3 inflammasome activation, pro-inflammatory cytokine release, and microglial activation-associated markers.

Amyloid-related and neurocellular outcomes were more heterogeneous. Some studies reported reduced Aβ burden or related pathological readouts after TLR4 inhibition or downstream pathway suppression, such as TAK-242 in APP/PS1 mice and Rac1 inhibition within the TLR4/Rac1/NLRP3 axis. In contrast, TLR4 loss-of-function in APP/PS1 mice reduced inflammatory activation but increased later Aβ deposition, fibrillar Aβ, and soluble Aβ42, indicating a dissociation between inflammatory suppression and amyloid handling. IAXO-101 further showed treatment- window-, sex-, and APOE genotype-dependent effects, with behavioral benefit not always accompanied by consistent Aβ-pathology reduction.

Functional outcomes showed the greatest heterogeneity. Beneficial effects were observed in selected contexts, including improved Morris water maze performance, prevention of Aβ oligomer-induced novel object recognition impairment, and rescue of *ex vivo* LTP impairment with RSLA. However, other studies reported limited, absent, or adverse behavioral translation, including worsened Morris water maze acquisition after TLR4 loss-of-function and limited spatial-memory rescue with TAK-242 in some settings. These findings indicate that behavioral consequences of TLR4 suppression are strongly dependent on model system, Aβ species, disease stage, intervention type, genotype, sex, and outcome domain.

**FIGURE 2:**
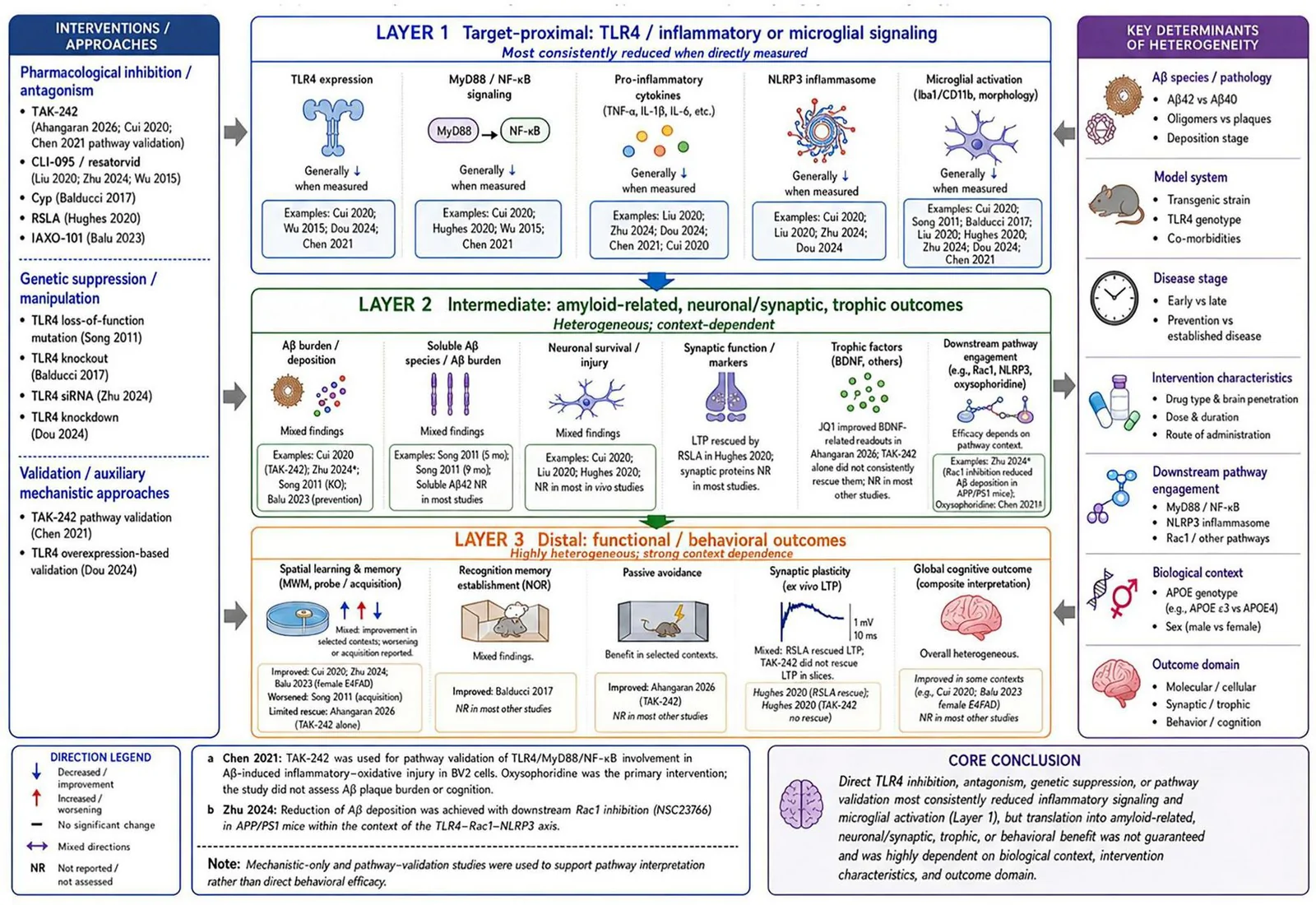
Hypothesis-generating evidence map of context-dependent inflammatory–amyloid–functional outcome dissociation after direct TLR4 inhibition, antagonism, genetic suppression, or pathway validation in AD-related models.

Overall, the available evidence supports a hypothesis-generating model of three-layer inflammatory–amyloid–functional dissociation, but it does not establish a uniform effect of TLR4 suppression. Higher-directness *in vivo* studies provided the main basis for cross-layer interpretation, whereas cellular, *ex vivo*, and pathway-validation studies primarily supported mechanistic plausibility. This conceptual synthesis is summarized in Figure 2.

### 3.5 Evidence directness and interpretative boundaries

Several interpretative boundaries were applied to avoid overstatement. First, not all studies directly measured canonical TLR4 downstream signaling; therefore, changes in selected inflammatory markers were not automatically interpreted as complete pathway suppression. Second, studies using Aβ as an experimental stimulus were not considered evidence for altered Aβ production, clearance, or plaque deposition unless these outcomes were directly assessed. Accordingly, findings from acute Aβ challenge or stimulation models were not interpreted as directly equivalent to findings from chronic APP/PS1 or EFAD transgenic models. Third, cellular tau-related protein changes in BV2 cells were not equated with neuronal tau pathology or neurofibrillary tangles. Finally, pathway-validation studies were distinguished from direct therapeutic-efficacy studies and were interpreted only as supportive evidence of biological plausibility.

Taken together, the observed inflammatory–amyloid–functional dissociation may reflect the context-dependent role of TLR4 in AD-related models: it may drive maladaptive neuroinflammation in some contexts while contributing to amyloid handling, immune surveillance, or disease-stage-dependent microglial function in others.

## 4 Discussion

### 4.1 Principal findings

This focused scoping review suggests that TLR4 suppression in AD-related models is associated with a layered, context-dependent pattern rather than a uniformly therapeutic effect. Across the 11 included studies, inflammatory or microglial readouts were most consistently attenuated, whereas amyloid-related, neuronal/synaptic, trophic, and behavioral outcomes were heterogeneous. When interpreted according to evidence directness, higher-directness *in vivo* studies provide the main support for cross-layer dissociation between inflammatory, amyloid-related, and functional outcomes, whereas cellular, *ex vivo*, and pathway-validation studies mainly support mechanistic plausibility. Overall, the available evidence is consistent with, but does not definitively establish, a three-layer inflammatory–amyloid–functional dissociation model: suppression of target-proximal inflammatory signaling does not necessarily translate into disease modification or functional recovery.

The most consistent finding was that TLR4 inhibition, antagonism, genetic suppression, or pathway validation reduced inflammatory-layer outcomes when these were directly measured. These included TLR4/MyD88/NF-κB-related signaling, NLRP3 inflammasome activation, pro-inflammatory cytokine release, oxidative inflammatory injury, and microglial activation-associated markers (Song et al., 2011; Liu et al., 2020; Cui et al., 2020; Chen et al., 2021; Dou et al., 2024; Zhu et al., 2024). This supports the role of TLR4 as an upstream regulator of AD-related innate immune activation, consistent with broader evidence linking TLR-mediated neuroinflammation to cognitive dysfunction (Calvo-Rodriguez et al., 2020; Squillace and Salvemini, 2022).

However, downstream consequences were less uniform. Some studies reported favorable effects on Aβ burden, neuronal injury, synaptic function, or memory. For example, TAK-242 reduced inflammatory signaling, Aβ plaque load, neuronal apoptosis-related readouts, and Morris water maze deficits in APP/PS1 mice (Cui et al., 2020). Similarly, CLI-095 or downstream Rac1 inhibition improved inflammatory and behavioral readouts within the TLR4/Rac1/NLRP3 axis (Zhu et al., 2024). In contrast, TLR4 loss-of-function reduced microglial activation but increased later Aβ deposition, fibrillar Aβ, soluble Aβ42, and Morris water maze impairment in APP/PS1 mice (Song et al., 2011). These findings suggest that TLR4 signaling is not purely detrimental and may, in selected contexts, contribute to amyloid handling or immune surveillance.

The APOE-stratified EFAD study further highlighted this context dependence. IAXO-101 produced the clearest inflammatory and memory-related benefits in female E4FAD mice, whereas effects were limited or absent in male E4FAD and female E3FAD mice. Its amyloid-related effects also differed between prevention and reversal paradigms (Balu et al., 2023). Thus, the net effect of TLR4 suppression appears to depend on biological context, including APOE genotype, sex, disease stage, and treatment window.

### 4.2 Interpretation of inflammatory–amyloid–functional dissociation

The observed inflammatory–amyloid–functional dissociation arises because inflammatory, amyloid-related, and functional outcomes are only partially coupled. At the inflammatory layer, TLR4 suppression can reduce cytokine release, NF-κB-related signaling, inflammasome activation, oxidative stress, or microglial reactivity. At the intermediate layer, however, Aβ deposition, soluble Aβ species, neuronal injury, synaptic function, and trophic factors may improve, remain unchanged, or worsen. At the distal layer, behavioral outcomes are even more heterogeneous.

This pattern challenges the assumption that reducing upstream inflammation is sufficient evidence of disease modification. Hughes et al. (2020) showed that Aβ42 oligomers induced TLR4/MyD88-dependent glial inflammatory responses, yet synaptic rescue depended on the antagonist and experimental compartment: RSLA rescued Aβ-induced LTP impairment, whereas TAK-242 did not rescue LTP in hippocampal slices. Similarly, Ahangaran et al. (2026) showed that TAK-242 reduced a CD86/CD163-based microglial polarization index but did not consistently rescue BDNF-related or spatial-memory outcomes; JQ1, rather than TAK-242, showed clearer effects on mature BDNF and spatial memory. These findings suggest that anti-inflammatory efficacy should not be equated with therapeutic efficacy.

Mechanistic-only studies support pathway interpretation but should not be used as standalone evidence of *in vivo* therapeutic efficacy. Wu et al. (2015) linked neuronal TLR4/NF-κB activation to amyloidogenic APP-processing readouts and Aβ1-42 secretion. Dou et al. (2024) showed that TLR4 knockdown reduced Aβ1-42-induced BV2 inflammatory, apoptotic, and tau-related cellular changes, but these findings should not be interpreted as neuronal tau pathology or *in vivo* cognitive benefit. Chen et al. (2021) supported TAK-242-sensitive TLR4/MyD88/NF-κB involvement in Aβ-induced BV2 inflammatory-oxidative injury, but oxysophoridine was the primary intervention and no Aβ plaque or behavioral outcome was assessed.

### 4.3 Sources of heterogeneity

Several factors may contextualize the divergent effects of TLR4 suppression across AD-related models. Because the included studies were methodologically heterogeneous and not suitable for quantitative subgroup analysis, these factors should be interpreted as plausible sources of heterogeneity rather than statistically confirmed modifiers of treatment effect. Nevertheless, considering these factors is important for understanding why attenuation of inflammatory signaling after TLR4 targeting did not consistently translate into amyloid-related, neuronal/synaptic, trophic, or behavioral benefit.

First, the included studies used different experimental systems, including transgenic AD mouse models, Aβ-injection models, BV2 and primary microglia, primary hippocampal neurons, astrocyte-related systems, hippocampal slices, and neuron-astrocyte co-cultures. These systems model different aspects of AD biology. Chronic APP/PS1 or EFAD models reflect progressive amyloid deposition and multicellular neuroimmune interactions, whereas acute Aβ-injection or Aβ-stimulation models mainly capture short-term amyloid-induced toxicity or inflammatory activation. *In vitro* models are useful for pathway dissection, but they cannot fully reproduce the spatial, temporal, vascular, and multicellular features of AD pathology. This limitation is particularly relevant for BV2 cells, whose transcriptional response to inflammatory stimulation may differ from that of primary microglia. Findings from BV2 cells, acute Aβ-stimulation systems, or acute Aβ challenge models should therefore be interpreted as model-specific mechanistic evidence rather than direct evidence of long-term amyloid modification, cognitive benefit, or *in vivo* disease modification (Hall and Roberson, 2012; Das et al., 2016; Jankowsky and Zheng, 2017).

Second, differences in Aβ species and preparation may have influenced the observed outcomes. Aβ1-42, Aβ25-35, soluble Aβ42 oligomers, fibrillar Aβ, and monomer-enriched Aβ preparations may differ in their capacity to activate TLR4-related signaling, induce NLRP3 inflammasome activation, affect phagocytosis, impair synaptic plasticity, or promote neuronal injury. Experimental conditions for Aβ oligomerization and fibrillogenesis can substantially alter biological activity, and soluble oligomeric Aβ has been widely discussed as a particularly synaptotoxic species. Therefore, TLR4 suppression may appear protective in acute oligomer-driven inflammatory models but less consistently beneficial in chronic amyloid-deposition contexts. Methodological differences in Aβ aggregation state should be considered when comparing studies using different Aβ preparations (Stine et al., 2003; Benilova et al., 2012; Reed-Geaghan et al., 2009; Balducci et al., 2017; Hughes et al., 2020).

Third, pharmacological inhibition should not be treated as equivalent to genetic suppression. TAK-242, CLI-095/resatorvid, Cyp, RSLA, and IAXO-101 differ in molecular target engagement, binding site, route of administration, pharmacokinetics, brain exposure, and pathway selectivity. TAK-242 selectively interferes with TLR4 intracellular-domain adaptor interactions, whereas other antagonists may act at different levels of ligand-receptor or co-receptor signaling. Genetic approaches, including TLR4 loss-of-function mutation, knockout, siRNA, knockdown, and overexpression-based validation, may also involve developmental compensation, long-term cell-state adaptation, or non-reversible pathway alteration that differs from acute or time-restricted pharmacological blockade. Therefore, pharmacological and genetic approaches were interpreted as distinct intervention categories rather than as interchangeable forms of “TLR4 inhibition.” These differences may help explain, but do not fully account for, why some studies reported benefit after pharmacological inhibition, whereas TLR4 functional loss was associated with impaired amyloid handling or worsened learning in other settings (Kawamoto et al., 2008; Matsunaga et al., 2011; Song et al., 2011; Cui et al., 2020; Hughes et al., 2020).

Fourth, disease stage and biological context may modify the consequences of TLR4 targeting. In some settings, TLR4 signaling may amplify cytokine release, NF-κB activation, inflammasome signaling, oxidative stress, and synaptic injury. In other settings, TLR4-related signaling may contribute to Aβ recognition, uptake, microglial recruitment, or immune surveillance. Evidence that TLR signaling participates in Aβ uptake and clearance, together with findings that TLR4 functional loss increased Aβ deposition in APP/PS1 mice, supports a cautious interpretation in which the role of TLR4 may vary according to amyloid context and disease stage (Tahara et al., 2006; Reed-Geaghan et al., 2009; Song et al., 2011; Doens and Fernández, 2014; Dallas and Widera, 2021).

Fifth, host biological modifiers should be considered. The EFAD study using IAXO-101 showed that TLR4 antagonism did not produce uniform effects across genotype and sex. The clearest inflammatory and memory-related benefits were observed in female E4FAD mice, whereas effects were limited or absent in male E4FAD and female E3FAD mice. Amyloid-related effects also differed between prevention and reversal paradigms. These findings suggest that APOE genotype, sex, and treatment window may be important modifiers of TLR4-targeted responses and should be prespecified in future studies rather than treated only as secondary explanatory variables (Balu et al., 2023).

Sixth, microglial-state classification may have influenced interpretation. Several studies used M1/M2-like markers or inflammatory polarization indices, but this binary framework is mainly a simplified reporting tool and is insufficient to capture AD-associated microglial heterogeneity. A reduction in inflammatory markers or M1/M2 ratio should therefore not automatically be interpreted as restoration of beneficial microglial functions such as Aβ clearance, synaptic support, metabolic adaptation, or trophic regulation. Future studies would benefit from integrating conventional inflammatory markers with functional and state-specific microglial measures, including phagocytosis, disease-associated microglial signatures, TREM2-APOE-related programs, and spatial or temporal microglial heterogeneity (Ransohoff, 2016; Colonna and Butovsky, 2017; Keren-Shaul et al., 2017; Krasemann et al., 2017; Deczkowska et al., 2018; Masuda et al., 2019).

Finally, outcome selection substantially influenced the interpretation of therapeutic direction. Studies focused mainly on cytokines, NF-κB signaling, oxidative stress, or NLRP3 activation tended to support TLR4 suppression as beneficial. In contrast, studies assessing Aβ deposition, soluble Aβ species, LTP, BDNF-related readouts, neuronal injury, or behavior revealed a more complex pattern. For example, Ahangaran et al. (2026) showed that TAK-242 reduced a CD86/CD163-based microglial polarization index but did not consistently rescue BDNF-related or spatial-memory outcomes, whereas JQ1 showed clearer effects on mature BDNF and spatial memory. This observation is biologically relevant because BDNF is closely linked to neuronal survival, synaptic plasticity, and long-term memory, and glia-neurotrophic factor interactions may influence neuroinflammation-related brain disorders. Similarly, Hughes et al. (2020) showed that glial inflammatory suppression and synaptic rescue were not fully coupled. Thus, heterogeneity across models, Aβ preparations, intervention types, biological modifiers, microglial states, and outcome domains provides a plausible framework for understanding why inflammatory suppression after TLR4 targeting does not consistently translate into amyloid-related or functional benefit (Hughes et al., 2020; Palasz et al., 2023; Gao et al., 2022; Ahangaran et al., 2026).

To avoid overinterpretation, these proposed sources of heterogeneity should be viewed as hypothesis-generating rather than conclusive. They highlight key variables that future studies should control, stratify, or directly test when evaluating TLR4-targeted interventions in AD-related models.

### 4.4 Implications for future TLR4-targeted strategies

These findings suggest that TLR4 should not be viewed simply as a harmful inflammatory receptor to be completely blocked. A more balanced interpretation is that TLR4 functions as a dual-role innate immune regulator. Excessive or sustained activation may drive maladaptive neuroinflammation, whereas context-appropriate TLR4 signaling may support amyloid recognition, immune surveillance, or microglial clearance functions (Song et al., 2011; Calvo-Rodriguez et al., 2020; Dallas and Widera, 2021; Squillace and Salvemini, 2022).

Future strategies should therefore emphasize selective modulation rather than indiscriminate inhibition. Timing, dose, route, duration, and brain penetration of TLR4-targeted agents should be aligned with disease stage and biological context. Sex- and APOE-stratified analyses are particularly important, especially in APOE4-related models. In addition, microglial outcomes should move beyond simplified M1/M2 descriptors and include phagocytosis, disease-associated microglial states, metabolic adaptation, synaptic support, and neurotrophic regulation (Jurga et al., 2020; Gao et al., 2023; Lepiarz-Raba et al., 2023).

The Ahangaran et al. (2026) study further suggests that receptor-level TLR4 inhibition and downstream transcriptional or neurotrophic modulation may not produce additive benefits. JQ1-mediated BRD4 inhibition improved memory and BDNF-related readouts, whereas TAK-242 alone did not consistently rescue spatial memory or mature BDNF signaling (Ahangaran et al., 2026). Thus, combined immune-epigenetic approaches should be interpreted carefully, particularly when suppression of upstream inflammatory signaling may interfere with compensatory neurotrophic or plasticity-related mechanisms.

Most importantly, future TLR4-targeted studies should move beyond inflammatory endpoints alone. Reduced cytokine release, NF-κB activation, or NLRP3 signaling is insufficient to establish disease modification. Robust evaluation should include amyloid production or clearance, neuronal and synaptic integrity, trophic support, and cognitive or functional recovery.

### 4.5 Limitations

Several limitations should be acknowledged. First, the number of studies directly evaluating TLR4 inhibition, antagonism, genetic suppression, or pathway validation in AD-related models remains limited. Second, heterogeneity in model system, Aβ species, intervention type, dosing, timing, sex, genotype, and outcome measurement precluded quantitative synthesis. Third, not all studies assessed all three outcome layers. Inflammatory endpoints were common, whereas amyloid-handling and behavioral endpoints were less consistently measured.

In addition, several studies used Aβ mainly as an experimental stimulus and did not directly assess Aβ production, uptake, clearance, or plaque deposition. Mechanistic-only, cellular, *ex vivo*, and pathway-validation studies were included to support pathway interpretation, but they should not be interpreted as direct evidence of amyloid modification, cognitive benefit, or *in vivo* therapeutic efficacy. Although a SYRCLE/CAMARADES-informed methodological appraisal was added for *in vivo* animal studies and a separate custom appraisal framework was used for *in vitro*, *ex vivo*, and cell-based studies, the appraisal was used to contextualize evidence confidence rather than to exclude studies or generate pooled estimates. Incomplete reporting of randomization, allocation concealment, blinding, sample-size justification, attrition, selective outcome reporting, Aβ preparation, intervention timing, and dose-exposure information across some studies limits the strength of the conclusions.

Another limitation is that the review protocol was not prospectively registered or deposited in a public repository. Although the main review elements were predefined before formal screening and detailed search, screening, and eligibility records are provided in the supplementary materials, the absence of a publicly accessible prespecified protocol limits independent verification of whether eligibility criteria, screening decisions, or outcome-layer definitions changed during the review process. Therefore, because this was a focused scoping review with a limited and heterogeneous evidence base, the present synthesis should be interpreted as a transparent but exploratory evidence map rather than as confirmatory or graded evidence of intervention efficacy.

## 5 Conclusion

The available preclinical evidence supports an exploratory and hypothesis-generating model of context-dependent inflammatory–amyloid–functional dissociation after direct TLR4 inhibition, antagonism, or genetic suppression in AD-related models, rather than a uniform therapeutic effect. Inflammatory readouts were relatively consistently attenuated when measured, whereas amyloid-related and functional outcomes were heterogeneous. These findings should not be interpreted as confirmatory evidence of therapeutic efficacy. Future studies should assess TLR4-targeted interventions using integrated inflammatory, amyloid-handling, and functional endpoints.
