# Supplementary material for: Direct toll-like receptor 4 inhibition in Alzheimer’s disease models: a focused scoping review of inflammatory, amyloid-handling, and functional outcomes
Source: Front Aging Neurosci. 2026 Aug 17;18:1889254. doi: 10.3389/fnagi.2026.1889254 (PMC13526921; doi:10.3389/fnagi.2026.1889254)
Supplement: Supplementary file 1 [file Table_1.docx]

Supplementary Table S1. PRISMA-ScR checklist.

| **PRISMA-ScR Item** | **Reported?** | **Location in manuscript** |
| --- | --- | --- |
| Title | Yes | Title page |
| Abstract | Yes | Abstract |
| Rationale | Yes | Introduction, paragraph 4 |
| Objectives | Yes | Introduction, final paragraph |
| Eligibility criteria | Yes | Methods 2.2 |
| Information sources | Yes | Methods 2.3 |
| Search | Yes | Supplementary Table S1 |
| Selection of sources | Yes | Methods 2.3; Figure 1 |
| Data charting process | Yes | Methods 2.4 |
| Synthesis of results | Yes | Methods 2.5 |
| Results of individual sources | Yes | Table 1 and Table 2 |
| Limitations | Yes | Discussion 4.5 |
| Protocol and registration | Yes | Methods 2.6; no registered protocol |
