# Supplementary material for: Direct toll-like receptor 4 inhibition in Alzheimer’s disease models: a focused scoping review of inflammatory, amyloid-handling, and functional outcomes
Source: Front Aging Neurosci. 2026 Aug 17;18:1889254. doi: 10.3389/fnagi.2026.1889254 (PMC13526921; doi:10.3389/fnagi.2026.1889254)
Supplement: Supplementary file 2 [file Table_2.docx]

**Supplementary Table S2. Complete database-specific search strategies**

| **Database** | **Search strategy** | **Search field** | **Time span** | **Limits** |
| --- | --- | --- | --- | --- |
| PubMed | ("Alzheimer Disease"[Mesh] OR Alzheimer* OR "Alzheimer's disease" OR AD)  AND  ("Toll-Like Receptor 4"[Mesh] OR TLR4 OR "toll-like receptor 4")  AND  (TAK-242 OR CLI-095 OR resatorvid OR "TLR4 inhibitor" OR "TLR4 antagonist" OR "TLR4 siRNA" OR "TLR4 knockdown" OR "TLR4 knockout" OR "TLR4 silencing" OR "TLR4 mutation")  AND  (amyloid OR Aβ OR "amyloid beta" OR "beta amyloid" OR microglia OR neuroinflammation OR NLRP3 OR inflammasome OR "NF-kappaB" OR "NF-κB" OR MyD88 OR Rac1 OR cognition OR memory OR "Morris water maze" OR synaptic OR neuron*) | Title/Abstract and MeSH | 2026/5/21 | No restriction on publication year; original experimental studies considered |
| Web of Science Core Collection | (Alzheimer* OR "Alzheimer disease" OR "Alzheimer's disease") AND (TLR4 OR "toll-like receptor 4" OR "toll like receptor 4") AND (TAK-242 OR CLI-095 OR resatorvid OR "TLR4 inhibitor*" OR "TLR4 antagonist*" OR "TLR4 siRNA" OR "TLR4 knockdown" OR "TLR4 knockout" OR "TLR4 silencing" OR "TLR4 suppression") AND (amyloid OR Abeta OR "A beta" OR "amyloid beta" OR "beta amyloid" OR microglia* OR neuroinflammation OR inflammation OR NLRP3 OR inflammasome OR "NF-kappaB" OR "NF kappa B" OR MyD88 OR Rac1 OR cognition OR cognitive OR memory OR learning OR "Morris water maze" OR behavior* OR synaptic OR neuron* OR neuroprotect*) | Topic | 2026/5/21 | Articles and original experimental studies |
| Scopus | ( Alzheimer* OR "Alzheimer's disease" OR "Alzheimer disease" )  AND  ( TLR4 OR "toll-like receptor 4" OR "Toll like receptor 4" )  AND  ( TAK-242 OR CLI-095 OR resatorvid OR "TLR4 inhibitor" OR "TLR4 inhibition" OR "TLR4 antagonist" OR "TLR4 blockade" OR "TLR4 suppression" OR "TLR4 siRNA" OR "TLR4 knockdown" OR "TLR4 knockout" OR "TLR4 deletion" OR "TLR4 deficiency" OR "TLR4 deficient" OR "TLR4 silencing" OR "TLR4 mutation" ) | Title, Abstract, Keywords | 2026/5/21 | Articles; original experimental studies considered |
| Embase | ('alzheimer disease'/exp OR alzheimer*:ti,ab OR "Alzheimer disease":ti,ab OR "Alzheimer's disease":ti,ab)  AND  ('toll like receptor 4'/exp OR TLR4:ti,ab OR 'TLR-4':ti,ab OR "toll-like receptor 4":ti,ab OR "toll like receptor 4":ti,ab)  AND  ('resatorvid'/exp OR resatorvid:ti,ab OR 'TAK-242':ti,ab OR 'TAK 242':ti,ab OR 'CLI-095':ti,ab OR 'CLI 095':ti,ab OR "TLR4 inhibitor":ti,ab OR "TLR4 antagonist":ti,ab OR "TLR4 siRNA":ti,ab OR "TLR4 knockdown":ti,ab OR "TLR4 knockout":ti,ab OR "TLR4 silencing":ti,ab) | Emtree and title/abstract | 2026/5/21 | Articles; original experimental studies considered |
