# Supplementary material for: Direct toll-like receptor 4 inhibition in Alzheimer’s disease models: a focused scoping review of inflammatory, amyloid-handling, and functional outcomes
Source: Front Aging Neurosci. 2026 Aug 17;18:1889254. doi: 10.3389/fnagi.2026.1889254 (PMC13526921; doi:10.3389/fnagi.2026.1889254)
Supplement: Supplementary file 3 [file Table_4.docx]

**Supplementary Table S4.** SYRCLE/CAMARADES-informed methodological appraisal and evidence-confidence assessment of the included studies.

| 、**No.** | **Study** | **Model relevance** | **Directness of TLR4 manipulation** | **In vivo validation** | **Cross-layer outcome coverage** | **Key methodological strengths** | **Main methodological limitations** | **Overall evidence confidence** |
| --- | --- | --- | --- | --- | --- | --- | --- | --- |
| 1 | **Song et al., 2011** | High; APPswe/PS1dE9 transgenic AD mouse model examined at disease-relevant stages. | High; genetic TLR4 loss-of-function mutation directly tested reduced TLR4 signaling. | Yes. | Strong; inflammatory/microglial, amyloid-related, and behavioral outcomes were assessed. | Provides key in vivo evidence linking reduced TLR4 signaling to decreased microglial activation but worsened later amyloid burden and MWM acquisition. | Genetic loss-of-function may involve developmental or compensatory effects; pharmacological reversibility and cell-specific TLR4 effects were not resolved. | **High / higher-directness paradox evidence** |
| 2 | **Wu et al., 2015** | Low to moderate; primary hippocampal neuron model, not an in vivo AD model. | Moderate; CLI-095 and PDTC used to probe TLR4/NF-κB signaling. | No. | Limited; inflammatory and APP-processing-related readouts only. | Supports a neuronal TLR4/NF-κB–APP-processing link. | LPS-driven neuronal activation may not fully model AD pathology; no microglial component, amyloid plaque assessment, behavior, or in vivo validation. | **Low / supportive mechanistic evidence only** |
| 3 | **Balducci et al., 2017** | Moderate to high; acute Aβ oligomer-induced mouse model with TLR4 knockout validation. | High; selective TLR4 antagonist and TLR4 knockout were used. | Yes. | Moderate; inflammatory and memory outcomes were assessed, but chronic amyloid handling was not. | Strong acute in vivo evidence that Aβ oligomer-induced glial activation and NOR impairment are TLR4-dependent. | Acute Aβ oligomer model does not assess chronic plaque burden, Aβ clearance, progressive AD pathology, or long-term intervention effects. | **Moderate to high / acute functional evidence** |
| 4 | **Cui et al., 2020** | High; APP/PS1 mice plus Aβ25–35-induced BV2 microglia. | High; TAK-242 used as a direct TLR4 inhibitor; NLRP3-siRNA used for pathway validation. | Yes. | Strong; inflammatory, amyloid-related, neuronal injury, and behavioral outcomes were assessed. | Integrates in vivo APP/PS1 evidence with in vitro pathway support; reports MWM, Aβ plaque load, inflammatory signaling, and neuronal apoptosis-related outcomes. | BV2 phagocytosis was bead-based rather than direct Aβ phagocytosis; M1/M2 markers may oversimplify microglial states. | **High / higher-directness beneficial-inhibition evidence** |
| 5 | **Liu et al., 2020** | Moderate; BV2 and primary microglia with HT-22 conditioned-medium neurotoxicity model. | High for cellular mechanism; CLI-095 directly inhibited TLR4 signaling in the experimental paradigm. | No. | Limited to moderate; inflammatory and indirect neuronal injury outcomes were assessed. | Provides mechanistic support for a TLR4/NLRP3-mediated microglial neurotoxicity pathway. | No animal validation, plaque assessment, APP processing, synaptic readouts, or behavioral outcomes. | **Low to moderate / supportive mechanistic evidence** |
| 6 | **Hughes et al., 2020** | Moderate; cellular, neuron–astrocyte co-culture, and hippocampal slice models using soluble Aβ42 aggregates. | High mechanistic directness; TAK-242, RSLA, and TLR4/MyD88 knockout systems were used. | No animal behavioral validation; ex vivo slice validation present. | Moderate; inflammatory, LTP, and cell-death outcomes were assessed. | Strong mechanistic-functional evidence linking soluble Aβ42 aggregates to TLR4/MyD88-dependent inflammatory signaling and ex vivo synaptic impairment. | No in vivo behavioral or amyloid-pathology evidence; LTP rescue differed by antagonist and experimental compartment. | **Moderate / supportive mechanistic-functional evidence** |
| 7 | **Chen et al., 2021** | Low to moderate; Aβ-stimulated BV2 microglial cell model. | Low to moderate for direct TLR4 therapy; TAK-242 was used for pathway validation, while oxysophoridine was the primary intervention. | No. | Limited; inflammatory–oxidative and BV2 viability outcomes were assessed. | Supports involvement of TLR4/MyD88/NF-κB signaling in Aβ-induced microglial inflammatory–oxidative injury. | Not a standalone TLR4-inhibitor efficacy study; no in vivo validation, amyloid-pathology endpoint, neuronal pathology, synaptic measure, or behavior. | **Low / supportive pathway-validation evidence only** |
| 8 | **Balu et al., 2023** | High; EFAD mice with APOE3/APOE4 and sex-stratified design. | High; IAXO-101 used as CD14/TLR4 antagonist. | Yes. | Strong; inflammatory, amyloid-related, and MWM outcomes were assessed. | Strong context-dependent in vivo evidence incorporating sex, APOE genotype, and prevention/reversal treatment windows. | Effects varied by sex, APOE genotype, and treatment window; amyloid-related effects were inconsistent across paradigms. | **High / context-dependent higher-directness evidence** |
| 9 | **Dou et al., 2024** | Low to moderate; Aβ1–42-induced BV2 microglial AD-like cell model. | High for cellular causality; lentiviral TLR4 knockdown and overexpression were used. | No. | Limited; inflammatory, apoptosis-related, and BV2 tau-related protein changes were assessed. | Provides bidirectional cellular evidence supporting TLR4-dependent BV2 inflammatory and apoptotic responses. | BV2 tau-related protein changes are not equivalent to neuronal tau pathology; no in vivo amyloid, neuronal, synaptic, or behavioral validation. | **Low / supportive genetic cell-model evidence only** |
| 10 | **Zhu et al., 2024** | High; Aβ1–42-treated BV2 microglia and APP/PS1 mice. | High; TLR4 siRNA, CLI-095, and Rac1 inhibition were used to interrogate the TLR4/Rac1/NLRP3 axis. | Yes. | Strong; inflammatory and behavioral outcomes were assessed; amyloid-related outcome was partly intervention-specific. | Integrates cellular pathway evidence with APP/PS1 in vivo MWM outcomes; supports a targetable Aβ–TLR4–Rac1–NLRP3 inflammatory axis. | Aβ deposition reduction should be interpreted specifically in relation to Rac1 inhibition; direct amyloid-lowering effects of CLI-095 should not be overgeneralized. | **High / higher-directness beneficial-inhibition evidence** |
| 11 | **Ahangaran et al., 2026** | Moderate to high; Aβ1–42-induced rodent model. | Moderate to high; TAK-242 tested alone and with JQ1, but canonical TLR4 downstream signaling was not fully quantified. | Yes. | Moderate; microglial polarization, BDNF-related markers, and behavior were assessed; amyloid pathology was not. | Provides in vivo evidence of dissociation between selected microglial polarization changes, neurotrophic markers, and spatial-memory outcomes. | Aβ plaque burden and Aβ clearance were not assessed; TLR4 expression, MyD88/NF-κB/NLRP3 signaling, and inflammatory cytokines were not fully quantified; JQ1 co-intervention complicates attribution. | **Moderate / higher-directness discordant evidence** |

**Note:** This table provides a narrative methodological quality and evidence-confidence assessment rather than a formal risk-of-bias score. Higher confidence was assigned to in vivo studies with direct TLR4 manipulation and cross-layer inflammatory, amyloid-related, and behavioral outcomes. Cellular, ex vivo, and pathway-validation studies were retained as supportive mechanistic evidence and were not interpreted as standalone therapeutic-efficacy evidence.
